# Lp(a), oxidized phospholipids and oxidation-specific epitopes are increased in subjects with keloid formation

**DOI:** 10.1186/s12944-022-01720-z

**Published:** 2022-11-01

**Authors:** Sundeep Ruder, Brett Mansfield, Andrew Ronald Immelman, Nissi Varki, Phuong Miu, Frederick Raal, Sotirios Tsimikas

**Affiliations:** 1 Carbohydrate & Lipid Metabolism Research Unit, Faculty of Health Sciences, University of the Witwatersrand, Johannesburg, South Africa; 2 Department of Pathology, University of California, San Diego, USA; 3 Sulpizio Cardiovascular Center, Division of Cardiovascular Medicine, University of California, 9500 Gilman Drive, 92093- 0682 San Diego, USA

## Abstract

**Background:**

Keloid formation following trauma or surgery is common among darkly pigmented individuals. Since lipoprotein(a) [Lp(a)] has been postulated to have a putative role in wound healing, and also mediates atherosclerotic cardiovascular disease, it was assessed whether Lp(a), its associated oxidized phospholipids and other oxidation-specific biomarkers were associated with keloid formation.

**Methods:**

This case-control study included darkly pigmented individuals of African ancestry, 100 with keloid scarring and 100 non-keloid controls. The lipid panel, hsCRP, Lp(a), oxidized phospholipids on apolipoprotein B-100 (OxPL-apoB), IgG and IgM apoB-immune complexes and IgG and IgM autoantibodies to a malondialdehyde mimotope (MDA-mimotope) were measured. Immunohistochemistry of keloid specimens was performed for both Lp(a) and OxPL staining.

**Results:**

Cases and controls were well matched for age, sex and lipid profile. Mean Lp(a) (57.8 vs. 44.2 mg/dL; *P* = 0.01, OxPL-apoB 17.4 vs. 15.7 nmol/L; *P* = 0.009) and IgG and IgM apoB-immune complexes and IgG and IgM MDA-mimotope levels were significantly higher in keloid cases. Keloid tissue stained strongly for OxPL.

**Conclusion:**

Darkly pigmented individuals of African ancestry with keloids have higher plasma levels of Lp(a), OxPL-apoB and oxidation-specific epitopes. The commonality of excessive wound healing in keloids and chronic complications from coronary revascularization suggests avenues of investigation to define a common mechanism driven by Lp(a) and the innate response to oxidized lipids.

**Supplementary Information:**

The online version contains supplementary material available at 10.1186/s12944-022-01720-z.

## Highlights

Keloid formation is common among individuals of African ancestry.

Lp(a) and OxPL are associated with acute and chronic CVD events.

Lp(a) and OxPL-apoB levels are elevated in patients with keloids.

Keloids express OxPL staining but not Lp(a) staining.

OxPL on Lp(a) may be mechanistically involved in keloid formation.

**Supplementary Information:**

The online version contains supplementary material available at 10.1186/s12944-022-01720-z.

## Introduction

Lipoprotein(a) [Lp(a)] is a cholesterol ester-rich low density lipoprotein (LDL)-like particle composed of a single apolipoprotein B100 (apoB) covalently linked to apolipoprotein(a) [1]. Epidemiological and genetic data has provided support for elevated levels of Lp(a) being a risk factor for both atherosclerotic cardiovascular disease (ASCVD) [2–4] and aortic stenosis [5, 6]. Lp(a) is postulated to mediate cardiovascular disease through 3 key mechanisms [7]: inflammation through its content of oxidized phospholipids (OxPL), anti-fibrinolytic effects through its apo(a) component [8] and atherogenicity via its LDL-like component.

A physiological role, if any, of Lp(a) remains undefined. Elevated Lp(a) levels are clearly associated with ASCVD and aortic stenosis, but there have been no other non-cardiovascular phenotypes noted [7]. Conversely, absence of Lp(a), which is rare, has not been associated with any known clinical adverse events [9, 10]. There have been several reports of a higher rate of incident diabetes in subjects with very low Lp(a) levels of < 5 mg/dL [11–13], which may represent 10% of the population at large [14], but it has not been established if these are causal or reverse causality effects of insulin resistance resulting in lower Lp(a) levels.

A putative physiological role of Lp(a) is to potentiate rapid wound healing or to prevent excessive bleeding [15]. These presumably occur at a young age or during childbirth and may provide the evolutionary pressure to maintain high levels among some individuals across populations. The apo(a) component of Lp(a) contains several lysine binding sites [16, 17], including a potent one on kringle IV_10_, (KIV_10)_ that allows apo(a) to bind to denuded endothelium and presumably deliver its cargo of cholesterol and other lipids for cell membrane repair. Indirect evidence for such a role of Lp(a) may be in keloid formation, representing an exaggerated wound healing effect. Keloids are common among darkly pigmented individuals. A previous study has shown that keloids are associated with carotid atherosclerosis but not explained by traditional cardiovascular risk factors [18]. Furthermore, Lp(a) is abundantly present in coronary bypass [19] and both elevated Lp(a) and OxPL in native coronary artery lesions [20] and have also been associated with both acute and long-term adverse outcomes in patients undergoing coronary artery bypass graft (CABG) surgery [21–23] or percutaneous coronary intervention (PCI) [24–26].

A potentially causal link between Lp(a) and the higher prevalence of keloids in this population group is postulated. The aim of this study was, therefore, to measure Lp(a) and their associated OxPL in darkly pigmented African patients with keloids and to determine whether there is any relationship between Lp(a) and keloid formation.

## Materials and methods

### Study subjects

Two hundred darkly pigmented subjects were recruited, one hundred with obvious keloid scarring and one hundred controls without evidence of keloid scarring. All participants were of black African ancestry, apart from one individual with a history of keloids who was of mixed ancestry. Subjects with keloids were identified at a designated “keloid clinic” run by the Department of Plastic Surgery, Faculty of Health Sciences, University of the Witwatersrand. Many of the keloids had developed following ear piercing or shaving and because of being located on the face the patients requested removal. The time between the injury and the onset of keloid scarring was not recorded. Black African control subjects of the same sex and within 5 years of age with previous surgery/trauma and no keloid development were enrolled.

### Laboratory variables

After informed consent 20mL blood was drawn from a cubital vein. Total cholesterol, triglycerides, high density lipoprotein cholesterol (HDL-C) calculated low density lipoprotein cholesterol (LDL-C), high sensitivity-C-reactive protein (hsCRP) and serum creatinine were measured using standard assays.

### Measurement of *lp(a)*, oxidized phospholipids on apolipoprotein B-100 (OxPL-apoB) and other oxidation-specific epitopes

Lp(a) [27], OxPL-apoB [28], IgG and IgM apoB-immune complexes [29] and IgG and IgM autoantibodies to a malondialdehyde mimotope (MDA-mimotope) [30] were measured by using in house chemiluminescent enzyme-linked immunosorbent assays (ELISA) developed at the University of California San Diego as previously described.

### Immunohistochemistry of keloid tissue

Details of immunohistochemical techniques to stain for the apolipoprotein(a) component of Lp(a) with murine monoclonal antibody LPA4, developed at UCSD [27], OxPL epitopes with murine monoclonal antibody E06 and apoB-100 with murine monoclonal antibody MB47 have been previously described [20, 29, 31]. In brief, keloid tissues were paraffin embedded, cut into 7 μm thick sections and mounted on charged slides. The sections were deparaffinized with Histoclear and rehydrated through graded ethanol. For antigen retrieval, sections were incubated with Sodium Citrate buffer (pH 6.0) in water bath at 95–100 °C for 20 min, then blocked with 5% normal goat serum/1% BSA/TBS for 30 min at room temperature. Monoclonal antibodies LPA4, E06 and MB47 diluted with blocking buffer to 5 µg/ml were used to stain sections in a humidified chamber at 4 °C overnight to detect apolipoprotein(a), OxPL and apoB-100, respectively. Sections were then incubated with an anti-mouse IgG-alkaline phosphatase (Sigma A3438) diluted with blocking buffer at 1:50 for 30 min at room temperature, and then visualized with Vector Red substrate (Vector SK-5100). Sections were counterstained with hematoxylin for 30 s and mounted with Simpo-Mount (IHCWorld EO3-18). Immunostaining of consecutive sections in the absence of primary Abs was used as a negative control. Images were captured with Hamamatsu Nanozoomer 2.0HT slide scanner with a 20X lens.

The methodology for immunostaining for apolipoprotein(a) is established and uses murine monoclonal antibody LPA4 that detects the 14-amino acid epitope TRNYCRNPDAEIRP present on KIV_5_, KIV_7_ and KIV_8_ of apolipoprotein(a), and also detects the partial sequence of NYCRNPDA present on KIV_2_. Furthermore, LPA4 has been used widely in the past and heavily stains apo(a) in coronary, carotid and aortic valve tissues [20, 31]. In addition, it was also used in the ELISA used to document the higher Lp(a) levels in subjects with keloids in this study.

### Statistical analyses

Data were analyzed using Statistica, version 13.3.0, June 2017, licensed through the University of the Witwatersrand. Standard descriptive statistics were used to describe the data for continuous variables (mean, median, range and standard deviation), and numbers and percentages were used for categorical variables. The Kolmogorov-Smirnov and Shapiro-Wilk tests were used to assess normality of the distribution of the data. For normally distributed data, 1-way ANOVA and unpaired Student t tests were used to compare differences between the groups, and for skewed data, the Mann-Whitney U test was used. Significance was defined as *P* < 0.05.

## Results

### Baseline characteristics

Table 1 displays the baseline characteristics of the study groups. There were no significant differences in mean age (27 vs. 29 years), sex (40% vs. 44% females) or weight (70.5 vs. 74.0 kg) between the cases and controls. Notably, there was a higher family history incidence of keloid in cases (13% vs. 0%) than controls. There were no significant differences in the lipid profiles or hsCRP, but cases had lower creatinine and higher eGFR than controls.

**Table 1:** Baseline characteristics of the study groups

Variable	Keloid cases (n = 100)	Controls (n = 100)	***P***-value
Age (years)	27 (22, 34.5)	29 (23, 37.5)	0.20
Females (%)	40	44	0.57
Weight (kg)	70.5 (60.0, 81.0)	74.0 (62.5, 85.5)	0.26
Family history of keloids (%)	13	0	< 0.0001
Smokers (%)	2	8	0.05
Previous cardiac event (%)	0	1	0.32
Total cholesterol (mmol/L)	4.12 ± 0.88	4.30 ± 0.89	0.15
HDL-cholesterol (mmol/L)	1.40 ± 0.36	1.40 ± 0.36	0.91
LDL-cholesterol (mmol/L)	2.48 ± 2.00	2.40 ± 0.79	0.78
Triglycerides (mmol/L)	0.99 ± 0.98	1.09 ± 0.60	0.03
hsCRP (mg/dL)	1.20 (0.57, 3.84)	1.28 (0.57, 3.58)	0.66
Creatinine (µmol/L)	65.2 ± 15.4	74.2 ± 16.4	< 0.0001
eGFR (mL/min/1.73m^2^)	138 (116, 166)	122 (103, 149)	< 0.0001

Data are presented as percentages (%), mean ± SD or median (IQR)

### Lp(a), oxidized phospholipids on apolipoprotein B-100 (OxPL-apoB) and other oxidation-specific epitopes

Table 2 displays the levels of Lp(a), OxPL-apoB and oxidation-specific biomarkers in cases and controls. Both baseline Lp(a) (normal < 30 mg/dL) and OxPL-apoB (> 75th percentile is > 7.5 nmol/L) levels were elevated in both keloid cases and controls. However, compared to controls, keloid cases had higher levels of Lp(a) (57.8 vs. 44.2 mg/dL; *P* = 0.01), OxPL-apoB (17.4 vs. 15.7 nmol/L; *P* = 0.009), IgG MDA-mimotope titers (607 vs. 422 RLU, *P* = 0.001), IgM MDA-mimotope titers (1512 vs. 1346 RLU, *P* = 0.001), IgG ApoB-IC titers (1291 vs. 888 RLU, *P* < 0.0001) and IgM MDA-mimotope titers (1404 vs. 814 RLU, *P* < 0.0001).

**Table 2:** Levels of Lp(a), OxPL-apoB and oxidation-specific biomarkers in cases and controls

Variable	Keloid cases (n = 100)	Controls (n = 100)	***P***-value
Lp(a) (mg/dL)	57.8 (34.7, 93.5)	44.2 (20.8, 72.3)	0.01
OxPL-apoB (nmol/L)	17.4 (14.3, 20.9)	15.7 (12.6, 19.3)	0.009
IgG MDA-mimotope, RLU	607 (408, 853)	422 (311, 639)	0.001
IgM MDA-mimotope, RLU	1512 (997, 2424)	1346 (857, 1923)	0.02
IgG ApoB-IC, RLU	1291 (916, 1764)	888 (691, 1184)	< 0.0001
IgM ApoB-IC, RLU	1404 (1010, 1997)	814 (574, 1185)	< 0.0001

Data represented as medians and interquartile ranges

### Immunohistochemistry of keloid tissue

**Fig. 1:**
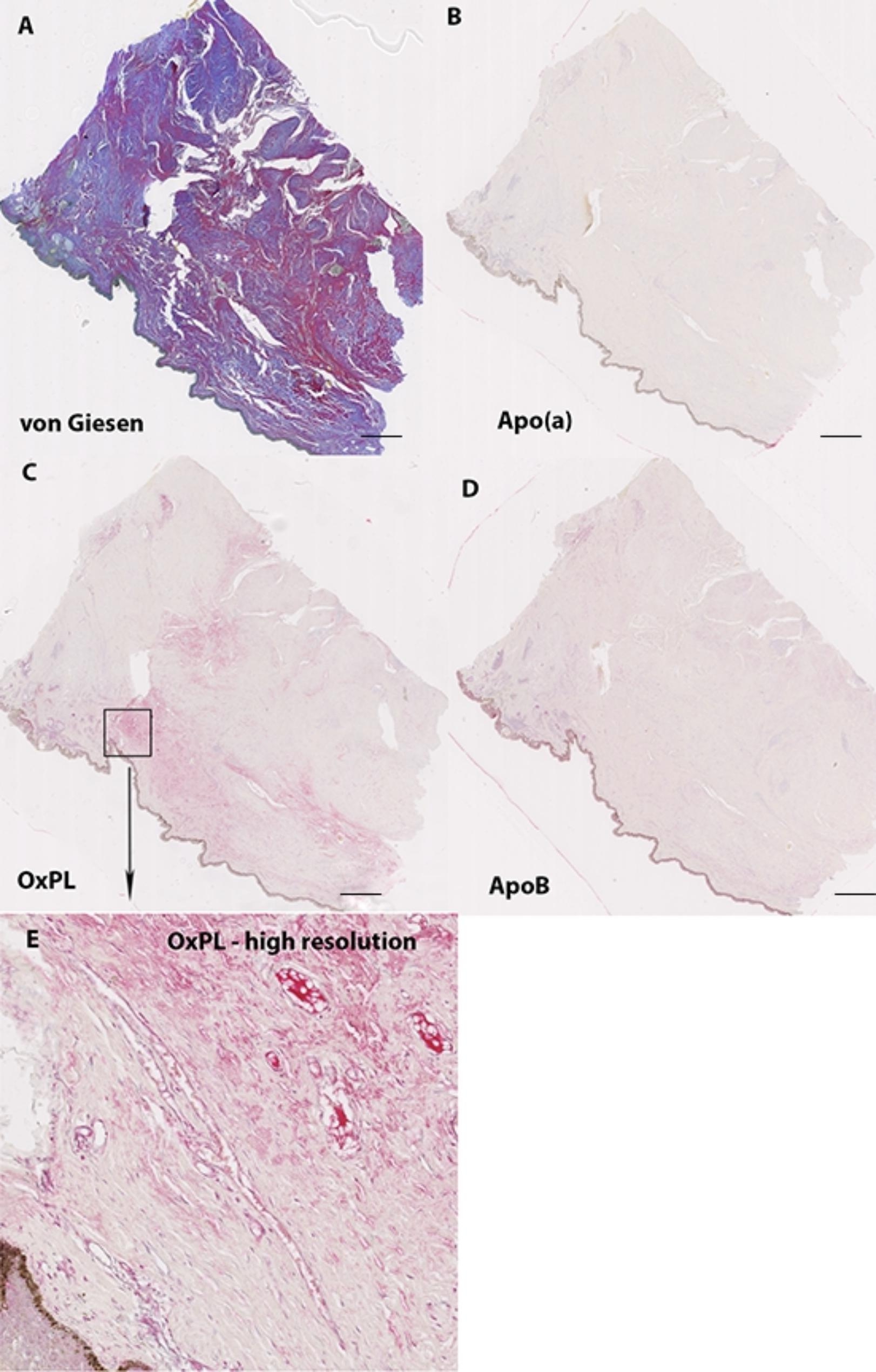
Representative sections of keloid immunostaining for Lp(a) and OxPL. Panel A represents von Giesen stain revealing significant amounts of collagen (red) in a highly cellular specimen (blue). Panel B represents immunostaining for apolipoprotein(a), which is not visible. Panel C represents immunostaining for E06-detectable OxPL (red) and panel E represents the inset at higher resolution. Panel D immunostaining for human apoB-100 (red). Bar = 500 μm

Representative sections of keloid immunostaining are shown in the Fig. 1. Staining of keloid tissue with von Giesen stain revealed significant amounts of collagen (Fig. 1, **panel A**, red) in a highly cellular specimen (blue). Interestingly, there was no evidence for the presence of apolipoprotein(a) in keloid tissue (Fig. 1, **panel B**). However, there was significant amount of E06-detectable OxPL (Fig. 1, **panel C**, red), which appeared to be in more cellular areas and less so in areas of abundant collagen. Very faint staining for human apoB-100 was also present (Fig. 1, **panel D**, red). A higher resolution image of the OxPL staining is shown in the Fig. 1, **panel E**. The no antibody control did not reveal any staining (not shown).

### Correlations among biomarkers

In the combined groups, Lp(a) was strongly correlated with OxPL-apoB (r = 0.79, *P* < 0.0001), and more modestly with IgG and IgM apoB-IC and MDA-mimotope (Table 3). The correlation coefficients were similar between keloid cases and non-keloid controls.

**Table 3:** Correlations between Lp(a) and laboratory variables

Variables	Combined groups (N = 200)	Non-keloid controls (n = 100)	Keloid cases (n = 100)
Total cholesterol	0.14 (0.16)	0.13 (0.21)	0.20 (0.046)
LDL-cholesterol	0.17 (0.09)	0.17 (0.08)	0.18 (0.08)
OxPL-apoB	0.79 (< 0.0001)	0.82 (< 0.0001)	0.75 (< 0.0001)
IgG MDA-Mimotope	0.26 (0.009)	0.27 (0.008)	0.22 (0.028)
IgM MDA-Mimotope	0.09 (0.34)	0.12 (0.22)	0.05 (0.64)
IgG ApoB-IC	0.04 (0.68)	-0.04 (0.70)	0.006 (0.95)
IgM ApoB-IC	0.09 (0.33)	0.03 (0.73)	0.03 (0.77)

Data given as r (*P*-value) for correlations with levels of Lp(a). Associations were tested using Pearson correlation with skewed variables first being normalised via log transformation or use of square roots (Lp(a)). No other variables correlated with Lp(a)

## Discussion

This study demonstrates that darkly pigmented African subjects with keloid formation had significantly higher levels of Lp(a), OxPL-apoB, circulating IgG and IgM apoB-immune complexes and MDA-mimotope levels compared to the non-keloid control group. Second, the presence of E06-detecatable OxPL within keloid scars was documented by immunostaining. The commonality of excessive wound healing in keloids and certain aspects of CABG and PCI that lead to vessel occlusion suggests avenues of investigation to define common mechanisms driven by Lp(a) and the innate response to oxidized lipids. It is estimated that 100 million patients develop scars in the developed world alone each year following surgery or trauma [32]. As opposed to excessive hypertrophic scar formation, keloids typically project beyond the original wound margins.

The prevalence of elevated Lp(a), defined as > 75 nmol/L ( ~ > 30 mg/dL) is approximately 20% of the population, or 1.5 billion people [33]. In this study, Lp(a) levels in both groups were higher than what is considered normal by most clinical laboratories. However, it is known that Lp(a) levels are 2–4 times higher in people of African descent compared to Caucasians [34, 35]. As opposed to most other racial/ethnic groups, darkly pigmented individuals of African descent tend to have higher population mean Lp(a) levels despite not necessarily having small isoforms, as documented in the Dallas Heart Study in US African Americans [35], suggesting additional unknown influences on apolipoprotein(a) expression and/or clearance. In the current study, despite the overall increased Lp(a) levels, the subjects with keloid formation had significantly higher Lp(a) levels. Keloid formation appears to be genetically determined as well, as suggested by the significant proportion of these subjects with keloids who also had a family history of keloid.

The higher circulating levels of OxPL-apoB in both groups may be related to the fact that the main lipoprotein carrier of OxPL, Lp(a), was also elevated. In prior studies, the 75th percentile for population cutoffs of OxPL-apoB are > 7.5 nmol/L [28, 36–38], but patients with elevated Lp(a) have significantly higher levels, often exceeding > 20 nmol/L [39, 40]. These findings were replicated in this study, but the subjects with keloid had even higher levels than the no-keloid controls. The findings on OxPL-apoB were complimented and consistent with the data on autoantibodies to MDA-mimotope and apoB-immune complexes, which generally reflect innate and adaptive immune activation awareness of the presence of pro-inflammatory oxidation specific epitopes. These biomarkers have often been associated with cardiovascular risk. For example, in epidemiological studies in stable patients, the IgG autoantibodies to oxidation-specific epitopes are positively associated with higher cardiovascular risk, but the IgM autoantibodies to oxidation-specific epitopes, which tend to be natural antibodies present at birth, seem to be protective [41]. In contrast, in more acute situations such as acute coronary syndromes and percutaneous coronary interventions, they tend to risk in parallel [27, 42]. The fact that both IgG and IgM biomarkers are higher in subjects with keloids suggests persistent immune activation to oxidation specific epitopes.

The immunohistochemistry data showed only faint apoB staining and could not identify the presence of apolipoprotein(a) in keloid tissue, suggesting that the Lp(a) holoparticle was not accumulating to any significant extent in the keloid tissue. In contrast, immunostaining for OxPL was present widely, but not necessarily strongest in areas of dense collagen deposition. Whether these OxPL are derived from the plasma or are generated by cells in situ within keloids cannot be determined from this study. In prior studies in mouse models which did not have the Lp(a) transgene or in cynomolgus monkeys which had Lp(a) but no associated OxPL due to variations in the KIV_10_ lysine binding pocket, OxPL could be documented to both accumulate and regress in response to high fat/cholesterol or regression diets, respectively [43, 44]. Finally, in human atheromata and aortic valve leaflets, Lp(a) and OxPL do necessarily always co-localize, suggesting that OxPL can be derived from additional sources besides Lp(a), such as apoptotic cells, cell membrane oxidation and other lipoproteins [20, 31].

There are additional clinical phenotypes that might be considered a response to injury, such as atherosclerosis, acute plaque rupture and restenosis in response to balloon angioplasty and stent placement, where Lp(a) may play a role [43, 45]. Recent studies suggest elevated Lp(a) levels are associated with chromic complications for PCI, both within the stent and adjacent to it, suggesting neoatherosclerosis. For example, a prospective single-center registry of 12,064 patients undergoing PCI showed that 31.1% of patients had Lp(a) levels > 30 mg/dL [24]. During a median follow-up of 7.4 years, the primary outcome, a composite of cardiovascular death, spontaneous myocardial infarction, and ischemic stroke, and repeated revascularization was significantly higher in the high Lp(a) group. Similarly, elevated Lp(a) and low molecular-weight apo(a) phenotype were independently associated with three-fold increase in risk of major adverse cardiovascular events within 15 years after CABG [23]. Mechanistically, the apo(a) component of Lp(a), which carries significant OxPL on KIV_10_, has been shown to stimulate smooth muscle cell proliferation and migration in a TGF-beta dependent process [46, 47]. These clinical phenotypes are considerably enriched in Lp(a)/OxPL, and specifically in advanced or ruptured plaques [20], chronic total occlusions [48], and in acute coronary syndromes [42]and PCI [27]. Direct evidence is also shown by the capture of plaque debris from carotid, coronary, renal and saphenous vein graft interventions, documenting the presence of OxPL [49].

## Strengths and limitations

This study is novel in that no other studies have examined the association between Lp(a) and keloid formation. A limitation of this study is that it is observational and cannot be used as evidence of causality. Additional studies are required to confirm and expand on these findings. Furthermore, apo(a) isoforms were not measured in this study and may have provided additional insights into the observations. Finally, whether these findings pertain to individuals who are lightly pigmented is not known and requires further study.

## Conclusion

In conclusion, darkly pigmented African individuals with keloids have higher plasma levels of Lp(a), OxPL-apoB and indirect biomarkers of oxidation-specific epitopes and higher levels of OxPL in keloid tissue. Given the relationship between raised Lp(a) and ASCVD, the presence of keloid formation may be an indicator of individuals at greater risk for ASCVD, however, these observations require additional study to determine whether a causal relationship exists.

## Electronic supplementary material

Below is the link to the electronic supplementary material.

Supplementary Material 1

## Data Availability

The datasets generated and/or analyzed during the current study are not publicly available due to confidentiality reasons but are available from the corresponding author on reasonable request.

## Abbreviations

OxPL: Oxidized phospholipids
Lp(a): Lipoprotein(a)
MDA: Malondialdehyde
ASCVD: Atherosclerotic cardiovascular disease
CABG: Coronary artery bypass graft
PCI: Percutaneous coronary intervention
IgG: Immunoglobulin G
IgM: Immunoglobulin M
ApoB-IC: ApoB immune complex
K: Kringles
eGFR: Estimated glomerular filtration rate
RLU: Relative light units
