# Supplementary material for: Lp(a), oxidized phospholipids and oxidation-specific epitopes are increased in subjects with keloid formation
Source: Lipids Health Dis. 2022 Nov 1;21:113. doi: 10.1186/s12944-022-01720-z (PMC9623907; doi:10.1186/s12944-022-01720-z)
Supplement: Supplementary file 1 — Supplementary Material 1 [file 12944_2022_1720_MOESM1_ESM.pdf]

Plagiarism Scan Report

Check Grammar | Make it Unique

Characters: 6060 **Words:** 836 **Sentences:** 20 **Speak Time:** 7 Min

100% Unique

0% Plagiarized

100%

Plagiarism Scan Report

Check Grammar | Make it Unique

Characters: 2744 **Words:** 392 **Sentences:** 20 **Speak Time:** 4 Min

94% Unique

6% Plagiarized

100%

Go Pro

View Plagiarized Sources

Plagiarism Scan Report

Check Grammar | Make it Unique

Characters: 4334 **Words:** 639 **Sentences:** 20 **Speak Time:** 6 Min

100% Unique

0% Plagiarized

100%

Plagiarism Scan Report

Check Grammar | Make it Unique

Characters: 6548 **Words:** 965 **Sentences:** 20 **Speak Time:** 8 Min

90% Unique

10% Plagiarized

100%

close
